# The Biosafety Research Road Map: The Search for Evidence to Support Practices in the Laboratory—*Bacillus anthracis* and *Brucella melitensis*

**DOI:** 10.1089/apb.2022.0042

**Published:** 2023-06-05

**Authors:** Stuart D. Blacksell, Sandhya Dhawan, Marina Kusumoto, Kim Khanh Le, Kathrin Summermatter, Joseph O'Keefe, Joseph Kozlovac, Salama Suhail Almuhairi, Indrawati Sendow, Christina M. Scheel, Anthony Ahumibe, Zibusiso M. Masuku, Allan M. Bennett, Kazunobu Kojima, David R. Harper, Keith Hamilton

**Affiliations:** ^1^ Mahidol-Oxford Tropical Research Medicine Unit, Faculty of Tropical Medicine, Mahidol University, Bangkok, Thailand.; ^2^ Centre for Tropical Medicine and Global Health, Nuffield Department of Medicine, Nuffield Department of Medicine Research Building, University of Oxford, Oxford, United Kingdom.; ^3^ Institute for Infectious Diseases, University of Bern, Bern, Switzerland.; ^4^ Ministry for Primary Industries, Wellington, New Zealand.; ^5^ United States Department of Agriculture, Agricultural Research Service, Beltsville, Maryland, USA.; ^6^ Abu Dhabi Agriculture and Food Safety Authority, Abu Dhabi, United Arab Emirates.; ^7^ Research Center for Veterinary Science, National Research and Innovation Agency, Indonesia.; ^8^ WHO Collaborating Center for Biosafety and Biosecurity, Office of the Associate Director for Laboratory Science, Center for Global Health, Centers for Disease Control and Prevention, Atlanta, Georgia, USA.; ^9^ Nigeria Centre for Disease Control and Prevention, Abuja, Nigeria.; ^10^ National Institute for Communicable Diseases of the National Health Laboratory Services, Johannesburg, South Africa.; ^11^ UK Health Security Agency, Porton Down, Salisbury, United Kingdom.; ^12^ Department of Epidemic and Pandemic Preparedness and Prevention, World Health Organization (WHO), Geneva, Switzerland.; ^13^ The Royal Institute of International Affairs, London, United Kingdom.; ^14^ World Organisation for Animal Health (OIE), Paris, France.

**Keywords:** *Bacillus anthracis*, *Brucella melitensis*, pathogen characteristics, biosafety evidence, biosafety knowledge gap

## Abstract

**Introduction:**

*Brucella melitensis* and *Bacillus anthracis* are zoonoses transmitted from animals and animal products. Scientific information is provided in this article to support biosafety precautions necessary to protect laboratory workers and individuals who are potentially exposed to these pathogens in the workplace or other settings, and gaps in information are also reported. There is a lack of information on the appropriate effective concentration for many chemical disinfectants for this agent. Controversies related to *B. anthracis* include infectious dose for skin and gastrointestinal infections, proper use of personal protective equipment (PPE) during the slaughter of infected animals, and handling of contaminated materials. *B. melitensis* is reported to have the highest number of laboratory-acquired infections (LAIs) to date in laboratory workers.

**Methods:**

A literature search was conducted to identify potential gaps in biosafety and focused on five main sections including the route of inoculation/modes of transmission, infectious dose, LAIs, containment releases, and disinfection and decontamination strategies.

**Results:**

Scientific literature currently lacks information on the effective concentration of many chemical disinfectants for this agent and in the variety of matrices where it may be found. Controversies related to *B. anthracis* include infectious dose for skin and gastrointestinal infections, proper use of PPE during the slaughter of infected animals, and handling contaminated materials.

**Discussion:**

Clarified vulnerabilities based on specific scientific evidence will contribute to the prevention of unwanted and unpredictable infections, improving the biosafety processes and procedures for laboratory staff and other professionals such as veterinarians, individuals associated with the agricultural industry, and those working with susceptible wildlife species.

## Introduction

The World Organization for Animal Health, World Health Organization (WHO), and Chatham House have collaborated to improve the sustainable implementation of laboratory biological risk management, particularly in low-resource settings. The Biosafety Research Roadmap project aims to support the application of laboratory biological risk management and improve laboratory sustainability by providing an evidence base for biosafety measures (including engineering controls) and evidence-based biosafety options for low-resource settings.

This will inform strategic decisions on global health security and investments in laboratory systems. This study involves assessing the current evidence base required for implementing laboratory biological risk management, aiming to provide better access to evidence, identifying research and capability gaps that need to be addressed, and providing recommendations on how an evidence-based approach can support biosafety in low-resource settings.

Here we present the general characteristics of *Bacillus anthracis* and *Brucella melitensis*, the current biosafety evidence, and available information regarding laboratory-acquired infections (LAIs) and laboratory releases.

## Materials and Methods

A 15 member technical working group (TWG) was formed to develop a Biosafety Research Roadmap (BRM) with the goal of supporting the application of laboratory biological risk management and improving laboratory sustainability by providing an evidence base for biosafety measures.

The TWG conducted a gap analysis for a selected list of priority pathogens on procedures related to diagnostic testing and associated research for those pathogens, including but not limited to sample processing, testing, animal models, tissue processing, necropsy, culture, storage, waste disposal and decontamination. To achieve this, the TWG screened databases, websites, publications, reviews, articles, and reference libraries for relevant data. The main research domains used to perform the literature searches were the ABSA database, Belgian Biosafety Server, CDC reports, WHO reports, PubMed, and internet searches for terms related to biosafety matters, including, for example, inactivation, decontamination, laboratory-acquired infections, laboratory releases and modes of transmission. The summary of evidence and potential gaps in biosafety was divided into five main sections: route of inoculation/modes of transmission, infectious dose, laboratory-acquired infections, containment releases, and disinfection and decontamination strategies.

## Bacillus anthracis

### General Characteristics

*B. anthracis* is the causative agent of anthrax. It is characterized as an anaerobic or facultatively anaerobic bacterium whose large gram-positive rods occur singly or in short chains. It is nonmotile, resistant to many antibiotics, and endospore forming. Endospore formation occurs in environmentally harsh conditions, including but not limited to drought and lack of nutrient material.

Anthrax is a zoonosis that spreads among herbivorous animals through contaminated soil and fodder. In omnivorous and carnivorous animals (i.e., canine, feline, and less frequently porcine species), the disease spreads through the consumption of or contact with contaminated meat, bone meal, and other fodder. *B. anthracis* is viable in soil and dried or processed hides.^1–3^

Anthrax is most common in agricultural regions where livestock is produced and is also found in areas populated by ungulates and similar types of herbivores (e.g., Africa, Asia, and the Middle East). It is far less common in industrial areas. It is an occupational hazard for veterinarians, ranchers, laboratorians, agriculture workers who handle infected animals, and workers who process hides, hair, wool, and bone products.^4,5^ *B. anthracis* also has the potential to be a biothreat agent.^6–8^

*B. anthracis* is a highly pathogenic agent, causing infections in four forms: cutaneous, inhalation, intestinal, and injection anthrax. Virulence is determined by the presence or absence of two plasmids: pXO1, which carries toxin genes, and pXO2, which has capsule genes. Both plasmids must be present to cause significant disease.^9–11^ It is classified by the U.S. Centers for Disease Control and Prevention (CDC) as a Tier 1 select agent, or “bioterrorism agent,” due to its stability, ability to cause fatal disease, ability to be aerosolized, and the potential for mass dissemination.

### Environmental Stability

*B. anthracis* endospores are uniquely stable in nature, being resilient to adverse environmental conditions. It can survive in soil and hides for many years or even decades. It is highly resistant to disinfectants and temperature fluctuations. However, irradiation, heat, hydrogen peroxide fumigation, calcium hypochlorite, chlorine, sodium hypochlorite, peracetic acid, formaldehyde, glutaraldehyde, sodium hydroxide, ethylene oxide, and chlorine dioxide (ClO_2_) are all effective disinfection and decontamination measures for *B. anthracis.*^11–13^

### Treatment and Prophylaxis

In the United States, the anthrax vaccine, BioThrax^®^ (Anthrax Vaccine Adsorbed), is approved by the Food and Drug Administration (FDA) and recommended for adults 18 through 65 years of age who are at risk of exposure to anthrax bacteria, including (1) laboratory workers who work with *B. anthracis*, (2) people who handle potentially infected animals or their carcasses, (3) certain military personnel, and (4) some emergency and other responders whose response activities might lead to exposure.^14^ People at risk should get three doses of the anthrax vaccine, followed by booster doses for ongoing protection.^14^

The anthrax vaccine series, in combination with antibiotic drugs, is also recommended as prophylaxis for (unvaccinated) people of all ages postexposure.^14^ Antibiotic treatment is usually oral ciprofloxacin or doxycycline; however, inhalational anthrax requires combination therapy with other antibiotics.^15^ Monoclonal antibody therapeutics and immune globulin therapy are also available.^15^

### Diagnosis

There are three clinical presentations of anthrax in humans: cutaneous (>95% of cases), orogastric, and inhalational. Diagnosis is straightforward if it is considered due to the signs and symptoms of the disease.^16^ The organism is readily observed by Gram or Wright stain in local lesions or blood smears and can be easily cultured from the blood and other body fluids.^16^ However, because of its rarity, anthrax is not often included in the differential diagnosis.

Unless there are occupational or lifestyle factors that may indicate exposure (i.e., laboratorian working with *B. anthracis*, veterinarian treating animals with signs or symptoms of disease, or individuals potentially exposed to susceptible animals or their products), the diagnosis of inhalation anthrax is rarely made until the patient is moribund, as initial symptoms present such as influenza and medical treatment are not sought until the person relapses with severe lung infiltration, difficulty breathing, and shock. Rapid diagnostic tests are under development and have been evaluated in the field to determine their accuracy and utility.^17–19^ Azure B microscopy is an accurate diagnostic test for animal anthrax for use in low-resource laboratory settings.^20^

## Biosafety Evidence

### Modes of Transmission

The most common routes of inoculation include exposure to infectious blood, skin lesion exudates, contact with hides, hair, wool, bone products, and bodily fluids and tissues from infected animals.^10,21^ Intravenous inoculation is also common among intravenous drug abusers.^22^

After inhalation, spores are initially ingested by the phagocytic cells lining the alveolar walls. These alveolar phagocytes detach from the wall and pass through the lymphatic vessels to the tracheobronchial glands. Spores were found in the tracheobronchial glands as early as 4 h and 6 min after intratracheal implantation.^10^

Booth et al reported the percutaneous route of infection in persons using injectable drugs, where a review of 27 confirmed cases of *B. anthracis* infection compared clinical findings in survivors and nonsurvivors.^23^ Although numerous cases of cutaneous anthrax have been attributed to work in tanneries and wool mills, a recent case of cutaneous anthrax infection was reported in a laboratory worker who assisted a coworker by transferring vials containing *B. anthracis* from the biosafety cabinet to the freezer in an adjacent room while not wearing gloves.^24^

The gastrointestinal route of infection has been demonstrated by Xie et al,^25^ who used a murine model of gastrointestinal (GI) anthrax infection and found that exposure to vegetative forms (after consumption of undercooked meat from recently slaughtered, infected animals) underlies infection in many, if not most, cases of GI anthrax in carnivores, including humans. Moreover, digestion in ruminants involves bacterial fermentation in the stomach, where vegetative *Bacillus* species are reported to grow.^25^

### Infectious Dose

In the case of inhalation, the median infective dose, ID_50_, is estimated to be between 8000 and 10,000 spores^26^ in humans. Rabbits are approximately three times more susceptible.^27^

### Laboratory-Acquired Infection

Anthrax is a rare LAI. The only recently documented case was in Texas in 2002 in an unvaccinated laboratory worker who handled vials containing *B. anthracis* specimens without wearing gloves.^28^ The vials were wiped with alcohol, not a sporicidal product. The patient was promptly treated and made a full recovery.

Two cases of intentional or accidental release from laboratories resulted in many instances.

1. In September 2001, a U.S. Army Research Institute for Infectious Disease civilian employee intentionally distributed anthrax spores through the U.S. Postal Service. Eleven people who came in contact with the infected mail were later diagnosed with inhalational anthrax, and five died from their illnesses.^29^ From October 4 to November 2, 2001, the U.S. CDC and state and local public health authorities reported 10 confirmed cases of inhalational anthrax and 12 confirmed or suspected cases of cutaneous anthrax in persons who worked in the District of Columbia, Florida, New Jersey, and New York.^30^
2. In 1979, human anthrax cases were reported in Sverdlovsk, USSR (now Ekaterinburg, Russia). Spores were accidentally released from a military biological weapons production facility^31^ with the official number of 96 cases reported,^32^ resulting in 66 fatalities occurring 1–4 days after the onset of symptoms.^29^

### Disinfection and Decontamination

#### Chemical

Rapid disinfection can be achieved with sodium hypochlorite (0.5%) containing vinegar on *B. anthracis* spores.^33^ *B. anthracis* spores were inactivated by 0.3% peracetic acid and 2% glutaraldehyde within 5 min and 6 h, respectively.^33^ Bleach Rite, a commercially available premixed stable formula of buffered (pH 12.3) 10% bleach, achieved a 6 log reduction in spores after 10 min of contact, whereas a 10% dilution of bleach and SporGon used at the manufacturers recommended concentration each required 20 min contact for complete inactivation.^34^ Sporicidin and Vesphene used at the manufacturer's recommended concentration did not achieve a log 6 reduction even after 60 min of exposure.^34^

#### Fumigation

Other known effective methods of spore inactivation include ozone gas, methyl bromide (MeBr), and heat inactivation. In the case of ozone gas, the materials in which the highest decontamination efficacy was achieved for *B. anthracis* spores were wallboard, paper, carpet, and wood with 6 log_10_ reduction (LR) occurring with 9800 ppm ozone, 85% relative humidity (RH), for 6 h. The laminate and galvanized metal materials were generally more difficult to decontaminate, requiring 12,000 ppm ozone, 85% RH, and 9–12 h contact time (CT) to achieve 6 LR of *B. anthracis.*^35^ MeBr with a concentration of 212 mg/L, the temperature at 27°C, and RH of 75%, with a CT of 24–36 h, is reported to be effective.^36^

The review of Rogers et al^37^ details numerous other historical studies using different exposure times and concentrations of fumigants for the inactivation of *B. anthracis* spores. Rogers et al^38^ describe how *B. anthracis* spores were susceptible to decontamination after exposure to 1000 ppm vaporized hydrogen peroxide for 20 min, 3000 ppm of ClO_2_ gas for 3 h, and 1.38–1.62 mL/ft^3^ of gaseous formaldehyde for 48 h.^37^

#### Thermal or autoclaving

Rapid-dry heat inactivation at higher temperatures (300–800°C) with a short CT (0.110 s) effectively inactivates *B. anthracis* spores.^39^ The WHO recommends autoclaving materials containing *B. anthracis* at 121°C for 1 h.^36^ All methods for disinfecting, decontaminating, and sterilizing contaminated materials should be validated as effective and revalidated if changes are made that could adversely impact effectiveness, including an increase in waste volume or different waste matrix, new equipment use, change in chemical used or supplier, or change in the process.

Evidence regarding the route of inoculation/modes of transmission, infectious dose, LAIs, and disinfection and decontamination strategies is provided in Table 1.

**Table 1.** **Detailed pathogen biosafety evidence for** *Bacillus anthracis*

Method	Details	Evidence (direct quote where available)	Reference	Evidence gap? (yes/no)
Route of inoculation	Inhalation	“Spores were found in the tracheobronchial glands as early as 4 hours and 6 minutes after intratracheal implantation.”	^10^	No
	Cutaneous	“Our review of 27 confirmed cases of *B. anthracis* infection in PWID compares clinical findings in survivors and non-survivors of this newly described form of infection.…Most survivors reported localized symptoms related to the injection site, and none required vasopressor therapy or mechanical ventilation. In contrast, most non-survivors had generalized symptoms and evidence of sepsis”	^23^	No
	Gastrointestinal	“We therefore contend that it is reasonable to assume that exposure to vegetative forms (after consumption of undercooked meat from recently slaughtered, infected animals) underlies infection in many, if not most, cases of GI anthrax in carnivores, including humans. Moreover, digestion in ruminants involves bacterial fermentation in the stomach, where vegetative Bacillus species are reported to grow [11]. For this reason, we believe that our murine model of GI anthrax infection parallels natural infection.”	^25^	No
Infectious dose	Inhalation 0.135 Deposited dose fraction 8000–10,000 spores	“We estimated the total deposited dose fraction is higher in humans than in rabbits: 0.135 for the human and 0.054 for the rabbit”	^27^	No
		“The results of KAMI indicated that the median infective dose, ID_50_, was between 8,000 and 10,000 spores”	^26^	No
	Cutaneous	No evidence		Yes
	Gastrointestinal	No evidence		Yes
	Injectional	No evidence		Yes
LAIs Laboratory Release-intentional Laboratory release	One case 2002, Texas Laboratory worker processed *B. anthracis* specimens	“On March 6, 2002, CDC's National Institute for Occupational Safety and Health (NIOSH) received a request for a health hazard evaluation from the director of Laboratory A to assist in the evaluation of a worker who had been diagnosed with cutaneous anthrax…The laboratory worker was one of three employees of Laboratory A who had primary responsibility for processing environmental *B. anthracis* specimens.”	^72^	No
	22 Cases (5 deaths) September 2001, DC	“In September 2001, a government employee of the US Army Research Institute for Infectious Disease intentionally distributed anthrax spores through the US Postal Service. Eleven people who came in contact with the infected mail were later diagnosed with inhalational anthrax, and 5 died from their illnesses.”	^29^	
		“From October 4 to November 2, 2001, the Centers for Disease Control and Prevention (CDC) and state and local public health authorities reported 10 confirmed cases of inhalational anthrax and 12 confirmed or suspected cases of cutaneous anthrax in persons who worked in the District of Columbia, Florida, New Jersey, and New York”	^30^	
	96 Cases April 1979, Sverdlovsk USSR (now Ekaterinburg, Russia) Spores released from military microbiology laboratory	“However, considerable interest in this outbreak was aroused by the suspicion that the epidemic was caused by release of spores from a military microbiology facility.”	^31^	
		“The official number of cases was only 96”	^32^	
		“66 died within 1 to 4 days following the onset of symptoms”	^29^	
Chemical inactivation	Disinfectant (spores) On tile plates, vinyl chloride: 0.5% sodium hypochlorite containing vinegar at 10°C On plywood plates: 1% and 5% sodium hypochlorite 0.3% peracetic acid, 5 min 2% glutaral, 6 h	“0.5% sodium hypochlorite containing vinegar on spores attached to vinyl chloride and tile plates. This rapid effect was also observed at 10°C. Sodium hypochlorite with a decreased pH due to the addition of vinegar has been shown to exhibit more marked sporicidal effects than sodium hypochlorite alone” “To inactivate *B. atrophaeus* spores attached to plywood plates, the plates were covered with gauze soaked in sodium hypochlorite at high concentrations (1% and 5%), 0.3% peracetic acid, or 2% glutaral. The spores were inactivated by 0.3% peracetic acid and 2% glutaral within 5 min and 6 h, respectively.”	^33^	No
	10% Bleach	“While Bleach Rite^®^ and 10% bleach reduce spore numbers by 90% within 10 minutes, a long contact time is required for complete disinfection. By contrast, although SporGon((R)) did not initially reduce the number of spores as quickly as Bleach Rite or 10% bleach, shorter contact times were required for complete eradication of viable spores.”	^34^	No
	Ozone (spores)—Complete inactivation Wallboard/paper 7000 ppm ozone, 85% RH, 4 h CT 9000 ppm ozone, 85% RH, 4/6/8 h CT 9800 ppm ozone, 85% RH, 6/9/12 h CT 12,000 ppm Ozone, 85% RH, 6/12 h CT Carpet 7000 ppm ozone, 85% RH, 8 h CT 9000 ppm ozone, 85% RH, 6/8/9/12 h CT 9800 ppm ozone, 85% RH, 6/9/12 h CT 12,000 ppm ozone, 85% RH, 9/12 h CT Wood 7000 ppm ozone, 85% RH, 8 h CT 9000 ppm ozone, 85% RH, 8 h 9800 ppm ozone, 85% RH, 6/9 h CT 12,000 ppm ozone, 85% RH, 6/12 h CT	“The LR results shown in bold in Tables 6 and 7 signify a test condition and material that resulted in complete inactivation” “Overall, ozone gas was effective in inactivating *B. anthracis* spores on all materials under at least one test condition”	^35^	No
	MeBr (spores) MeBr concentration 212 mg/L Temp 27°C RH 75% CT 24–36 h	“For example, under the fumigation conditions using 212 mg/liter MeBr, 27°C, and 75% RH, we report that 36 h was needed to achieve *a* ≥ 6-LR on all materials on the basis of the test 9 results, but the actual contact time needed would more likely be between 24 and 36 h”	^36^	No
Gaseous decontamination	≥1000 ppm hydrogen peroxide gas for 20 min	“*Bacillus anthracis, B. subtilis, and G. stearothermophilus* spores were dried on seven types of indoor surfaces and exposed to > or = 1000 ppm hydrogen peroxide gas for 20 min. Hydrogen peroxide exposure significantly decreased viable B*. anthracis, B. subtilis,* and *G. stearothermophilus* spores on all test materials except *G. stearothermophilus* on industrial carpet. Significant differences were observed when comparing the reduction in viable spores of B. anthracis with both surrogates. The effectiveness of gaseous hydrogen peroxide on the growth of biological indicators and spore strips was evaluated in parallel as a qualitative assessment of decontamination. At 1 and 7 days postexposure, decontaminated biological indicators and spore strips exhibited no growth, while the non-decontaminated samples displayed growth”	^38^	No
	ClO_2_ gas concentration 3000 ppm; 3 h; 70% RH; 22–24°C	“Fumigation testing maintaining a target ClO_2_ gas concentration of approximately 3,000 ppm for 3 hours promoted the inactivation of recoverable *B. anthracis* Ames spores from porous and nonporous indoor surface materials inoculated with approximately 1 x 10^8^ spores, corresponding to calculated log reductions ranging from 7.1 to 7.9”	^37^	No
	Vaporized formaldehyde 1.38 to 1.62 mL per cubic foot (0.40–0.46 per cubic meter)	“Young et al (1970) treated a textile mill contaminated with *B. anthracis* spores by vaporizing a 37% solution of formaldehyde in a steam cleaning machine and introducing into the sealed buildings at final concentrations ranging from 1.38 to 1.62 mL per cubic foot in which the temperature was above 26°C and relative humidity near saturation. Following the two-day treatment, surface sampling results showed a significant reduction in *B. anthracis* viable contamination”	^37,73^	No
Thermal inactivation	Rapid-dry heat inactivation 300–800°C 0.1–10 s	“Compared with common laboratory dry heating studies, temperatures in this study are much higher (from 300°C to 800°C as opposed to 120°C), and the exposure times are significantly shorter (0.1 to 10 s versus 30 to 120 min [6])”	^39^	No

CDC, Centers for Disease Control and Prevention; CT, contact time; LAI, laboratory acquired infection; RH, relative humidity; MeBr, methyl bromide.

## Knowledge Gaps

### Infectious Dose

The primary knowledge gap for *B. anthracis* is that the human infectious dose for contracting anthrax through inhalation, cutaneous, injection, and gastrointestinal routes is unknown. Doses, as is the case for inhalation, are typically modeled or extrapolated from animal studies and are often provided in terms of a lethal dose, not an infectious dose. Infectious doses are typically provided as ID_50_ and may overestimate the number of organisms needed to cause infection in more susceptible populations, including individuals with suppressed immune responses, other underlying conditions, and variations in health in general. This knowledge is important in informing the risk of an LAI through different routes of exposure and across populations, some of which may have increased susceptibility to infection.

### Most Appropriate Personal Protective Equipment and Contaminated Material Disposal Procedures During and After Necropsies in Low-Resource Settings

The government of Western Australia recommends using (personal protective equipment [PPE]) that can be discarded or held in a biosecure location pending test results and includes overalls, eye protection, facemask, disposable gloves, and impervious boots.^40^ However, there is a lack of standardized advice regarding the most appropriate PPE to use when performing a necropsy of an anthrax-suspected animal in low-resource settings bearing in mind that access to PPE may be limited.

Waste disposal depends on the type of waste generated and local capabilities. It may include hypochlorite solutions with 10,000 ppm available chlorine, autoclaving solid waste and PPE at 121°C for 60 min, and incineration of carcasses.^41^ There remains an opportunity to clarify and provide standardized advice on the disposal of contaminated materials (including carcasses) and PPE after animal necropsies in low-resource settings such that the environment is not contaminated with anthrax-containing materials.

## Conclusions

Most human anthrax infections result from handling infected animals or carcasses.^42^ Veterinarians are advised to wear appropriate PPE when handling suspected specimens and dispose of waste correctly. Necropsy and sampling should be conducted to the minimal extent required to reduce exposure risk, and nonessential personnel should be restricted from the necropsy area to avoid further contamination. LAIs in the microbiology laboratory are rare and have been attributed to the cutaneous route in recent years. A risk assessment should be conducted to determine appropriate PPE to be used in diagnostic and research laboratories, but at a minimum should include gloves, eye protection, and a laboratory coat or other form of covering for clothing.

## Brucella melitensis

### General Characteristics

Members of the genus *Brucella* (i.e., *B. melitensis, Brucella abortus*, *Brucella suis*, and *Brucella canis*) are zoonotic pathogens capable of infecting an extensive host range, including humans, cattle, swine, goats, sheep, deer, caribou, elk, dogs, and coyotes.^43^ Endemicity of brucellosis is worldwide^43^ and is generally considered an occupational disease for farm workers, abattoir workers, veterinarians, and tannery workers.

Bacteria of the *Brucella* genus are gram-negative coccobacilli of the Proteobacteria phylum. They are nonmotile, do not have a capsule, and cannot sporulate.^43^ *Brucella* spp. are classified by the CDC as “bioterrorism agents” due to their stability, ability to cause mass disease with a low infectious dose, ability to be aerosolized, and potential for mass dissemination. *Brucella* spp. can survive long periods in the environment, freezing, and thawing, and living for up to 4 months in milk, urine, water, feces, and damp soil. They are classified as a Risk Group 3 pathogen.^43,44^ Their relevance for biosecurity is high^43–46^ as there is potential for bioweapon use.

### Treatment and Prophylaxis

The prophylaxis/treatment for *Brucella* spp. infections include antibiotic therapy with streptomycin and doxycycline (streptomycin for 2–3 weeks and doxycycline for 8 weeks), gentamicin plus doxycycline (gentamicin for 5–7 days and doxycycline for 8 weeks), and tetracyclines.^43,47–49^ Eight weeks treatment regimen of two antibiotics has a high therapeutic success and reduced likelihood of relapse. Treatment may take weeks to months as the organism can be released periodically from abscesses it forms in the body. There are currently no licensed *Brucella* vaccines for use in humans.

### Diagnosis

Laboratory procedures include in vitro culture isolation and serological tests such as the Rose Bengal test, serum tube agglutination test, *Brucella* microagglutination test (BMAT), Coombs' test, enzyme-linked immunosorbent assay, and complement fixation. *Brucella* DNA is directly detected using PCR.^47,50,51^

## Biosafety Evidence

### Modes of Transmission

Common routes of infection include ingestion of contaminated raw milk or milk products; cutaneous or percutaneous transfer through direct contact with infected animal tissue, including aborted fetuses and bodily fluids; and inhalation of bacteria from contaminated materials.^43–45,52–55^ Slaughterhouse workers, veterinarians, and farmers are at the most significant risk of infection since mucosal and cutaneous/percutaneous transfer occurs by bacterial inoculation into mucus membranes and through cuts and abrasions of the skin while handling infected animal parts.^49,56–58^ *Brucella* spp. are also easily aerosolized, remain stable (and virulent) for a protracted period, and thus are easily transmitted through airborne routes and frequently induce disease through inhalation.^49,56–58^

### Infectious Dose

The infectious dose for *B. melitensis* is extremely low, between 10 and 100 aerosolized organisms.^49,57,59^ There are no literature reports regarding the infectious dose for cutaneous, percutaneous, mucosal, and oral exposure.

### Laboratory-Acquired Infections

*Brucella* spp. remain some of the most commonly reported LAIs^43–46,49,52,54,60^ and were in the top percentile of LAIs worldwide between 1979 and 2015, where brucellosis was reported as causing 378 LAIs.^61^ In an early report by Pike,^60^ 4079 LAIs caused by 159 biological agents were reported, with 10 pathogens causing 50% of all infections (brucellosis, Q fever, hepatitis, typhoid fever, tularemia, tuberculosis, dermatomycoses, Venezuelan equine encephalitis, psittacosis, and coccidioidomycosis).^60^ Before 1976, there were 423 cases of LAIs caused by *Brucella* spp. resulting in 5 deaths.^43^

In an extensive review of LAIs between 1979 and 2015, Byers and Harding reported that *Brucella* spp. (378), *Mycobacterium tuberculosis* (255), arboviruses (222), *Salmonella* spp. (212), *Coxiella burnetii* (205), Hantavirus (189), Hepatitis B virus (133), *Shigella* spp. (88), human immunodeficiency virus (48), and *Neisseria menigitidis* (43) accounted for 1753 of the 3230 LAIs reported.^46^ *Brucella* spp. infections resulted from the untreated exhaust released from a veterinary vaccine plant in Spain that infected 17.1% of the 164 employees; the attack rate was 39.5% for staff working in areas with open windows above the exhaust.^62^

### Environmental Stability

The environmental stability of *Brucella* spp. is variable and dependent on environmental conditions.^63–65^ Cooler environmental temperatures and reduced exposure to sunlight favor the survival of *Brucella* spp.^65^

### Decontamination and Inactivation

It has been reported that inactivation of *Brucella* spp. is commonly achieved through commercial disinfectants, detergents, temperature extremes (pasteurization), or steam.^39,44,50,66^

#### Chemicals

Effective disinfectants include iodophor, phenolic, chloramine, or hypochlorite.^43,48–50,67^ Concentrations of Lysol (phenolic) at 10 g/L, sodium hypochlorite at 2 g/L, and NaOH at 10 g/L were effective at inactivating brucellae in saline and soil at room temperature with recommended exposure times of 30, 20, and 10 min, respectively. Only sodium hypochlorite and NaOH retained efficacy when brucellae were in feces.^62^ Alkaline disinfectants have excellent efficacy against *Brucella* spp. even in organic matter.^62,67,68^ Sodium hypochlorite (bleach) and sodium hydroxide (lye) are preferred in dirty conditions or at low temperatures.^68^

These have been suggested as ideal disinfectants for brucellosis prevention and control, with lye preferred for animal housing environments and field disinfection. Bleach is preferred for laboratory, biological material, medical supplies, and smooth surface disinfection.^67^ Buffered neutral formalin (10% concentration) was highly effective in inactivating *Brucella* spp. bacteria within 4 h from tissue sections with high colonization levels.^69^ This may be most relevant for conducting laboratory work, that is, prepping and examining tissue sections.

#### Fumigation

*B. suis* inoculated on solid nonporous surfaces can be inactivated by exposure to 230 ppm of hydrogen peroxide vapor (VHP) for 5.5 h.^70^ The use of VHP would have to be validated for use on more porous materials such as concrete or wood and different *Brucella* species.

#### Thermal and autoclaving

Heat inactivation is effective, although the temperature plays a vital role in the time taken for complete elimination. With viable *Brucella* bacteria (*B. abortus*, *B. suis*, and *B. melitensis*), temperatures approaching boiling allowed complete inactivation to be achieved within 30–60 min; however, lower temperatures require much longer heating times (hours).^69^ Autoclaving at 121°C for at least 15 min can decontaminate tools exposed to the *B. canis.*^71^ Autoclave parameters should be validated for different matrices and different *Brucellae* species.

Evidence regarding the route of inoculation/modes of transmission, infectious dose, LAIs, and disinfection and decontamination strategies is provided in Table 2.

**Table 2.** **Detailed pathogen biosafety evidence for** *Brucella melitensis*

Method	Details	Evidence (direct quote where available)	Reference	Evidence gap? (yes/no)
Route of inoculation	Subcutaneous/percutaneous transfer	“Direct contact through skin abrasions with infected animal tissues (as in slaughterhouse workers) is also implicated…”	^49^	No
		“Transmission of brucellosis to humans occurs…through direct contact with infected animal parts (such as the placenta by inoculation through ruptures of skin and mucous membranes)”	^58^	
		“Transmission to humans occurs through opened skin exposed to animal secretions…, inoculation into the conjunctival sac…”	^57^	
		“Percutaneous infection through skin abrasions or by accidental inoculation has frequently been demonstrated.”	^56^	
		“Common routes of infection include inoculation through cuts and abrasions in the skin or via the conjunctival sac of the eyes…”	^74^	
	Inhalation	“…is airborne transmission. Brucella can be easily aerosolized, and when in air, can be easily transmitted through the airways and induce disease, while staying for a protracted period in this virulent form.”	^49^	No
		“Transmission of brucellosis to humans occurs…through the inhalation of infected aerosolized particles”	^58^	
		“Transmission to humans occurs through…infected aerosols…” “Laboratorians have acquired brucellosis by inhalation.”	^57^	
		“Inhalation of aerosols containing the bacteria, or aerosol contamination of the conjunctivae, is another route.”	^56^	
		“Common routes of infection include…inhalation of infected aerosols…”	^74^	
Infectious dose	Inhalation 10–100 microorganisms	“…10 to 100 aerosolized organisms are needed to cause disease…”	^57^	No
		“…small inoculum needed to induce human disease, traditionally described in the levels of 10–100 microorganisms.”	^49^	
		“As few as 10–100 bacteria may cause disease when inhaled.”	^59^	
	Cutaneous	No evidence		Yes
	Percutaneous	No evidence		Yes
	Oral	No evidence		Yes
	Ocular	No evidence		Yes
LAIs	Top percentile of LAIs worldwide	“*Mycobacterium tuberculosis*, *Coxiella burnetii*, hantaviruses, arboviruses, hepatitis B virus, *Brucella* spp., *Salmonella* spp., *Shigella* spp., hepatitis C virus, and *Cryptosporidium* spp. accounted for 1074 of the 1267 infections”	^46^	No
		“4079 LAIs were caused by 159 biological agents, although ten agents caused infections accounting for 50% of cases (brucellosis, Q fever, hepatitis, typhoid fever, tularemia, tuberculosis, dermatomycoses, Venezuelan equine encephalitis, psittacosis, and coccidioidomycosis)”	^46^	
		“Brucellosis is one of the main causes of LAIs and between 1979 and 2015, brucellosis was reported as causing 378 LAIs”	^61^	
	Survey of 23 laboratories (up to Biosafety level 4) Two cases of *Brucella melitensis*	“Only four of the 23 surveyed laboratories reported 15 LAIs caused by four different pathogenic organisms. Bacterial infections predominated, particularly biosafety level 3 bacteria belonging to the following species: *Mycobacterium tuberculosis* (ten cases), *Coxiella burnetii* (two cases), and *Brucella melitensis* (two cases)”	^75^	No
	71 LAIs found from global literature review (1982–2007)	“In the 28 laboratory exposure case reports, 167 workers were potentially exposed to Brucella spp., 71 (43%) of whom developed LAB”	^45^	No
	LAI reports literature review (1982–2016) Asia Pacific region Three cases of *Brucella* spp.	“A total of 27 LAI reports were published between 1982 and 2016…The most commonly reported pathogens causing LAIs were dengue virus (3 reports), severe acute respiratory syndrome coronavirus (SARS-CoV) (3 reports), *Brucella* spp. (3 reports) …”	^76^	No
	BSAT TLR reports in United States, 2004–2010 received by CDC Four cases *B. melitensis*	“There were 11 laboratory acquired infections (LAIs) that resulted from the incidents described in the 639 release reports received between 2004–2010. These LAIs were associated with exposures to *Brucella melitensis* (4 cases), *B. suis* (2 cases), *Francisella tularensis* (4 cases) and *Coccidioides immitis/posadasii* (1 case)”	^77^	No
	*Brucella* spp. at a veterinary vaccine plant in Spain the attack rate was 39.5% for staff	“An outbreak of acute brucellosis infection was detected among the employees of a biologicals manufacturing laboratory located in Girona, Spain. A clinical and epidemiologic investigation conducted among the 164 employees found 22 patients with clinical symptoms and positive serology, and six patients detected by serology only (attack rate: 17.1 per cent). Employees working in areas with open windows above the laboratory air extracting system had an attack rate of 39.5 per cent, substantially higher than those working in other locations. When vaccine was manufactured again, an electric oven reaching 300 degrees C had been installed in the air extracting system just before its exit to the exterior. Appropriate culture medium plates were exposed to the laboratory air before and after passing through the oven.”	^62^	No
Inactivation	Heat inactivation	“In our experiment, complete elimination of viable *Brucella* bacteria (*B. abortus*, *B. suis*, and *B. melitensis*) within 30 to 60 minutes required temperatures approaching boiling, whereas lower temperatures required much longer heating times (hours)” “Heat inactivation appears to be highly influenced by temperature with heating to near 100°C required for rapid killing of all bacteria within samples”	^69^	No
	Sodium hydroxide/bleach	“Sodium hypochlorite and sodium hydroxide are preferred with dirty conditions or at low temperatures. Actually, the two disinfectants are often selected due to its lower price and low toxicity”	^68^	Yes
		“In general, the present results suggested that in the process of brucellosis prevention and control, sodium hydroxide is preferred for animal housing environment and field disinfection, and sodium hypochlorite is preferred for laboratory, biological material, medical supplies, and smooth surface disinfection.”	^67^	
	Hypochlorite solutions	“Sodium hypochlorite and sodium hydroxide are preferred with dirty conditions or at low temperatures. Actually, the two disinfectants are often selected due to its lower price and low toxicity”	^68^	Yes
		“…should be washed down with an approved disinfectant (hypochlorite, iodophor or phenolic disinfectant at recommended working strength)”	^48^	
		“In general, the present results suggested that in the process of brucellosis prevention and control, sodium hydroxide is preferred for animal housing environment and field disinfection, and sodium hypochlorite is preferred for laboratory, biological material, medical supplies, and smooth surface disinfection.”	^67^	
	Phenolic disinfectants	“Phenolic disinfectants were also highly effective in quickly inactivating *Brucella* in solutions”	^69^	Yes
		“…should be washed down with an approved disinfectant (hypochlorite, iodophor or phenolic disinfectant at recommended working strength)”	^48^	
		“it may be concluded that all these disinfectant types including aldehydes, halogens, quaternary ammonium compound, phenolics, and alkalines could be selected for disinfection to prevent brucellosis”	^67^	
	Nano disinfectants	“By trying of some types of Nano disinfectants to evaluate its efficacy against *B. melitensis* the result was as following; the effect of Dettol and Glutaraldehyde was increased when combined with silver-NPs while calcium-NPs had lower effect especially with presence of organic matters” “Nano disinfectants had good reduction rate at low temperature even with presence of organic matters specially Glutaraldehyde with silver-NPs and Dettol with silver-NPs which had the highest reduction rate”	^68^	Yes
	Alkaline disinfectants	“Alkaline disinfectants as…have excellent efficacy against *Brucella* spp. even in presence of organic matters”	^68^	Yes
		“…bacterial efficacy of alkaline disinfectants solution was not comparable with the average results of three commercial farm disinfectants but treatment of up to 1:20 dilution of alkaline disinfectant solution was sufficient to exert bactericidal activity”	^78^	
		“…it may be concluded that all these disinfectant types including aldehydes, halogens, quaternary ammonium compound, phenolics, and alkalines could be selected for disinfection to prevent brucellosis”	^67^	
	Iodophor/halogens/quaternary ammonium compound	“…it may be concluded that all these disinfectant types including aldehydes, halogens, quaternary ammonium compound, phenolics, and alkalines could be selected for disinfection to prevent brucellosis”	^67^	Yes
		“…should be washed down with an approved disinfectant (hypochlorite, iodophor, or phenolic disinfectant at recommended working strength)”	^48^	
	10% Neutral formalin	“Buffered neutral formalin (10% concentration) was highly effective in inactivating *Brucella* bacteria by 4 hours from tissue sections that had high levels of colonization”	^69^	No
	Aldehydes	“it may be concluded that all these disinfectant types including aldehydes, halogens, quaternary ammonium compound, phenolics, and alkalines could be selected for disinfection to prevent brucellosis”	^67^	No
Thermal	Electric oven at 300°C added to veterinary vaccine manufacturing plant exhaust	Appropriate culture medium plates were exposed to the laboratory air before, and after passing through the oven.	^62^	No

BSAT, biological select agent and toxin; LAI, laboratory-acquired infection; SARS-CoV, severe acute respiratory syndrome coronavirus; TLR, theft, loss or release.

## Knowledge Gaps

The main evidence gaps are the agreed-upon working concentrations for many chemical disinfectants, including sodium hypochlorite, aldehydes, iodophors, halogens, and quaternary ammonium compounds.

In addition, the infectious dose for *Brucella* spp. through cutaneous, percutaneous, oral, and mucosal routes is unknown and important for informing the risk assessment in laboratory workers, veterinarians, and other professionals that may work with organisms or come in contact with infected animals or animal tissues.

## Conclusions

*Brucella* LAIs continue to be the most frequently reported LAIs even today. Therefore, all manipulations and handling of pathogenic *Brucella* spp. and diagnostic specimens suspected of containing them should not be processed on open work benches but should be carried out using Biosafety Level 3 (BSL-3) practices, primary containment equipment (e.g., biological safety cabinets), and facilities that can contain an aerosol in case of a spill. A thorough risk assessment should be conducted to inform the type of PPE required (to include respiratory protection) and the secondary containment features of the facility where work is conducted.

Factors that are used in the risk assessment should consist of agent-related information, the activities performed, the concentration of agent worked with, volume and matrices of materials handled, and procedures and equipment to best mitigate the risks. There is a lack of consistent information regarding working concentrations and CTs for many chemical disinfectants used routinely in laboratories. The efficacy of disinfectants should be validated against the differing contaminated matrices and the concentration required and exposure time.
